# Comparison of the Biomechanical Stability of Two Fix-and-Replace Techniques in an Acetabular Fracture Model with Pelvic Discontinuity

**DOI:** 10.3390/jcm15041419

**Published:** 2026-02-11

**Authors:** Nicholas A. Beckmann, Raphael S. Ernst, Stefan Jakobs, Martin Müller, Hannes Kuttner, This Aebi, Johannes D. Bastian

**Affiliations:** 1 Center for Orthopaedics, Traumatology and Spinal Cord Injury, University of Heidelberg, 69118 Heidelberg, Germany; 2 Department of Orthopaedic Surgery and Traumatology, Inselspital, Bern University Hospital, University of Bern, 3012 Bern, Switzerlandjohannes.bastian@insel.ch (J.D.B.); 3 RMS Foundation, 2544 Bettlach, Switzerland; 4 Department of Emergency Medicine, Inselspital, Bern University Hospital, University of Bern, 3012 Bern, Switzerland; 5 DePuy Synthes, 4528 Zuchwil, Switzerland

**Keywords:** acetabular fracture, traumatic pelvic discontinuity, Osteosynthesis, Ganz reinforcement ring, Burch-Schneider, fix-and-replace

## Abstract

**Background/Objectives:** Managing acetabular fractures remains a surgical challenge, particularly in cases involving traumatic pelvic discontinuity (PD). The optimal method for achieving primary stability is unclear, and biomechanical evidence comparing established techniques is limited. The goal of this biomechanical study is to evaluate if a Ganz reinforcement ring with the addition of a posterior-column plate and anterior-column screw (GRP) provides stability comparable to a Burch-Schneider reinforcement ring (BSR) with an additional anterior- and posterior-column screws construct. **Methods:** The primary biomechanical stability of two acetabular “fix-and-replace” techniques—BSR versus GRP—using standardized 4th-generation Sawbones^®^ hemipelvis models with T-type fractures (PD) was compared. Relative 3D micromotions at the fracture site (Zone 1: Posterior-column; Zone 2: Anterior-column; Zone 3: Oblique to transverse fracture, and Zone 4: Ischiopubic ramus) were measured under increasing cyclic loading (100 cycles per load level) at 200 N, 400 N, 800 N, and 1200 N using an optical motion tracking system. A detected fracture gap of 1000 µm or more during/after the cyclic load was defined as fixation failure. **Results:** Fixation failure was not observed in any of the six artificial hemipelves with treated (3 BSR, 3 GRP) T-type acetabular fractures. Under cyclic, increasing load (200–1200 N), the mean fracture gap remained small at 200 N and 400 N with no significant differences between techniques. At 800 N, GRP fixation showed a non-significant increase in micromotion. At 1200 N, significantly greater displacements were observed in Zones 2–4 with GRP compared to BSR (*p* < 0.005), whereas no difference was found in Zone 1 (*p* = 0.424). Modelled slope and intercept comparisons confirmed a significantly steeper increase in fracture gap with GRP in zones 2–4 at higher loads (≥800 N, *p* < 0.01) while remaining under 1000 µm. **Conclusions:** Both fixation methods demonstrated sufficient construct stability without catastrophic failure, with minimal displacement (<1 mm) and with no significant difference in stability at the posterior column.

## 1. Introduction

Acetabular fractures, which in the most severe cases can present as pelvic discontinuity (PD), are potentially life-changing incidents that can lead to prolonged pain, functional disability and diminished quality of life [1]. In Germany the incidence of acetabular fractures was reported as 12/100,000 persons in 2019 with a 58% increased incidence since 2009 [2]. Acetabular fractures including PD have a bimodal distribution, occurring in the 20–30 age group because of high-energy trauma (motor vehicle accidents) and in older adults mainly because of low-impact trauma such as a fall from a walking height. The incidence in older adults is anticipated to increase as a result of changing demographics [3].

The ultimate goal of treatment is restoration of normal pelvic anatomy and preservation of the native hip joint with diminished potential for the later development of post-traumatic osteoarthritis. This ideal can be achieved in young patients with good bone quality and healing potential who are able to benefit from a range of operative techniques. However, this goal is often unachievable for some older adults, particularly female patients with moderate to severe osteoporosis/osteopenia due to ageing. These patients have limited osseointegration capacity and are at high risk of complications arising from lengthy periods of immobility following open reduction and internal fixation (ORIF) alone [4,5]. The choice of treatment regime is determined by patient age and comorbidity in addition to fracture characteristics. For osteoporotic patients, cementless techniques are usually not selected to restore hip function.

Acute PD represents a complete separation of the pelvic bone through the acetabulum, as found with fractures involving both columns such as T-shaped fractures. Historically, treatment options for acetabular fractures have included non-operative management for non-displaced fractures, ORIF, which has long been considered the gold standard [6], and more recently, total hip arthroplasty (THA), either as a standalone procedure or in combination with ORIF [7]. The optimal treatment strategy for acetabular fractures, particularly in older patients, remains a matter of debate. However, achieving fracture stability and minimizing micromotion at the bone–implant interface are critical prerequisites for the successful fixation of the acetabular component. This biomechanical study investigates the primary stability of two fixation constructs that may serve as viable treatment options for this high-risk patient population.

We hypothesize that a reinforcement ring combined with a posterior-column plate and an anterior-column screw (GRP) provides primary stability comparable to that achieved with a BSR cup construct with additional anterior- and posterior column screws.

## 2. Materials and Methods

To test the hypothesis, we evaluated and compared the primary stability of a standardized T-type acetabular fracture with PD (according to the Judet–Letournel classification [8]) when treated with the two constructs. Primary stability was assessed by measuring the fracture gap under cyclic loading conditions simulating the moderate weight-bearing forces encountered during normal gait [9,10,11].

This was achieved by measuring the gap along the fracture lines when subjected to loads simulating the moderate weight-bearing load experienced during normal gait [9,10,11] (see below).

**Figure 1:**
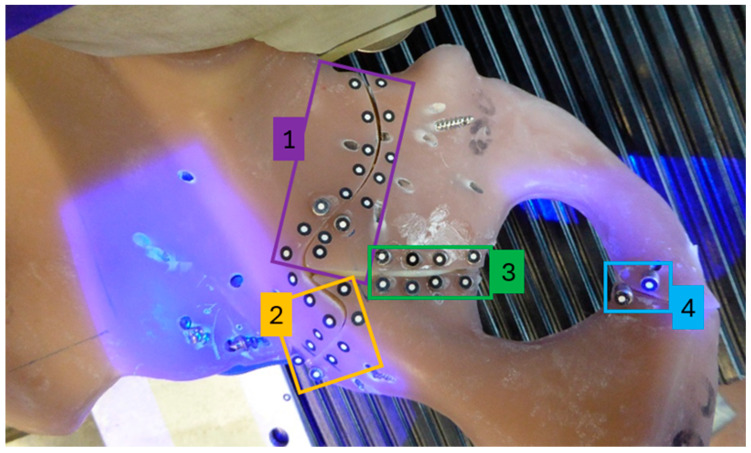
View of the hemipelvis from medial looking toward the quadrilateral surface. Left is the iliac crest, bottom is anterior, right is the os ischium, and top is the posterior aspect with iliac spine. This photograph is of the 4th Generation Sawbones^®^ with fracture lines, divided into four zones: zone 1 (Posterior Column, orange) and zone 2 (Anterior Column, purple) are along the posterior and anterior transverse fracture gap, respectively, zone 3 (in green) is along the fracture between anterior and posterior column, and zone 4 (in blue) is along the fracture of the ischiopubic ramus.

Six hemipelves specimens (4th Generation Sawbones^®^ Europe AB, Malmo, Sweden) each had a mechanically created standardized T fracture by means of a CAD-assisted jet cutter (Sigla-Lorenz Siegenthaler, Grenchen, Switzerland) that uses water with abrasive particles under pressure (>200 MPa) analogous to [12]. This technique allowed the consistent and precise creation of the desired fracture in a standard composite bone. The Sawbone^®^ was chosen to provide reproducibility and standardization in this in vitro scenario, and it is a proven specimen having been used for decades of biomechanical research [13,14,15]. At present, to our knowledge, no standardized or validated biomechanical model exists for osteoporotic pelvic discontinuity, and cadaveric models with predefined acetabular osteoporosis are logistically challenging, costly, and poorly reproducible. The fracture lines were divided into four zones: Zone 1 and zone 2 were along the posterior and anterior transverse fracture gap, respectively, zone 3 was along the fracture between anterior and posterior column and zone 4 was along the fracture of the ischiopubic ramus (Figure 1).

**Figure 2:**
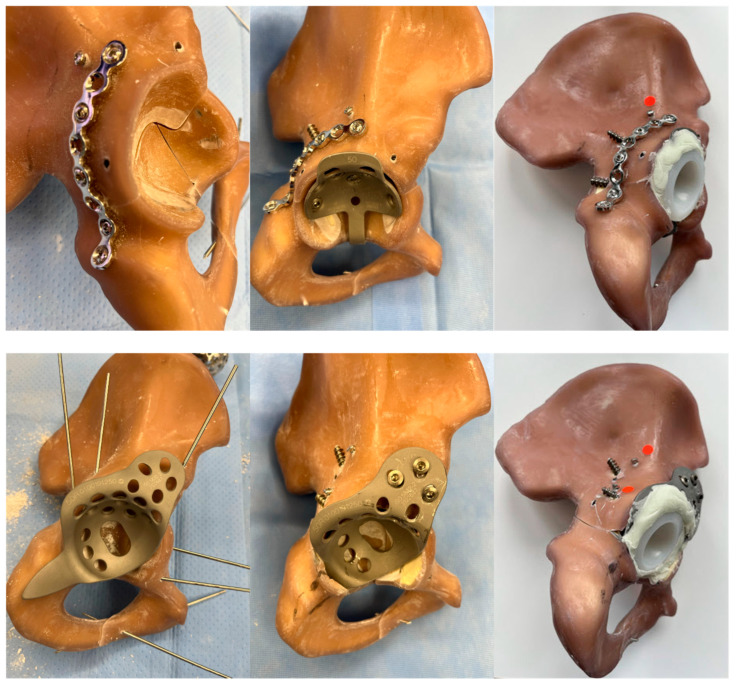
Photographs from posterolateral of the two constructs during and after implantation. Top: shows GRP (or Reinforcement Ring with PC plate and AC screw) after plating (**left**), after Ganz ring implantation (**centre**), and after cementation of the cup (**right**); Bottom: shows BSR (or Burch-Schneider Reconstruction with PC screw and AC screw) with ring placed on top of acetabulum (**left**), ring inserted and screwed into pelvis (**center**), and after cementation of the cup (**right**). Red dots indicate either AC or PC screw heads.

The six hemipelves were divided into two groups and treated using different surgical techniques. Three specimens were reconstructed with the GRP and three with the BSR (see Figure 2). Cemented acetabular fixation was used in this study, consistent with clinical practice in osteoporotic bone. All procedures were performed by a fellowship-trained senior consultant orthopedic surgeon with >10 years of experience in acetabular and revision hip surgery using the original instrumentation provided by the manufacturers.

1. Reconstruction with Ganz reinforcement ring with posterior column plating and anterior column screw (GRP) placement using a 3D printed guide: A 3.5/4.5 mm pelvic plate (Stryker; Selzach, Switzerland) was fitted across the posterior column fracture with five 3.5 mm screws. Additional 3.5 mm cortical screws were placed into the anterior and posterior column. Afterwards the acetabulum was reamed with spherical reamers (Zimmer Biomet; Winterthur, Switzerland) up to size 54, and a 50 mm GRP (reinforcement ring with hook, Zimmer Biomet) was fitted into the acetabulum with five 6.5 mm cancellous bone screws (Zimmer Biomet). For the ORIF combined with a Ganz acetabular reinforcement ring and supplementary plate fixation, one 40 mm, one 125 mm, one 65 mm, one 50 mm, one 36 mm, and one 28 mm screw (all 3.5 mm diameter) for fixation were used. Afterwards a 48/32 mm Durasul^®^ low-profile cemented cup 48/32 (Zimmer Biomet) was cemented into the cage with an inclination of 45 degrees and an anteversion of 15 degrees using Optipac 40 Bone cement (Zimmer Biomet), which was achieved using a standardized mechanical alignment jig and verified clinically as is done in the intraoperative setting as well as with a goniometer.

2. Reconstruction with Burch-Schneider reinforcement ring and an anterior- and a posterior column screws (BSR) using a 3D printed guide: The acetabulum was reamed with spherical reamers (Zimmer Biomet) up to size 54. A BSR cage size 50 (Burch-Schneider™, reinforcement cage, Zimmer Biomet) was placed into the acetabulum, and the superior flange was adapted to the os ilium. Five 6.5 mm fully threaded cancellous bone screws (Zimmer Biomet) were placed into/through the cage and three through the superior flange. A 3.5 mm cortical screw was placed into the anterior and posterior columns. Burch-Schneider cage construct was fixed using two 40 mm screws for fixation of the cage itself, one 130 mm screw, and one 95 mm screw (all 3.5 mm diameter). Finally, a 48/32 mm Durasul low-profile cup (Zimmer Biomet) was cemented to the cage as described above.

### 2.1. Test Setup

**Figure 3:**
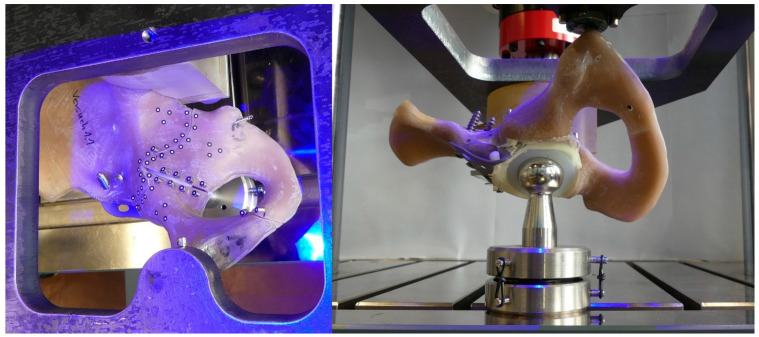
(**Left**): Top view of the optical markers located beneath the steel plate, mounted to the load cell of the testing machine. (**Right**): Bottom view of the test setup, showing the replicated prosthetic femoral head resting on the machine table, allowing free movement within the plane.

Each hemipelvis specimen was placed in a previously made 3D negative imprint of the sacroiliac joint, which allowed firm fixation of the hemipelvis to the sacroiliac joint (see Figure 3). The 3D negative imprint was screwed to a thick steel plate. This steel plate also allowed the support of the symphysis on a polished and lubricated steel surface, the screw connection to the load cell of the testing machine and a view onto the fracture lines. The symphysis was fixed in only one degree of freedom, which still allowed for planar movement and rotation of the symphysis to better mimic physiologic fixation as described in previous studies [16,17,18]. A replicated hip prosthetic head (32 mm) was positioned on the machine table, manufactured to match the previously described implants. This part of the test setup was placed on two sliding surfaces with roller bearings in between to achieve a low-friction and planar movement.

**Figure 4:**
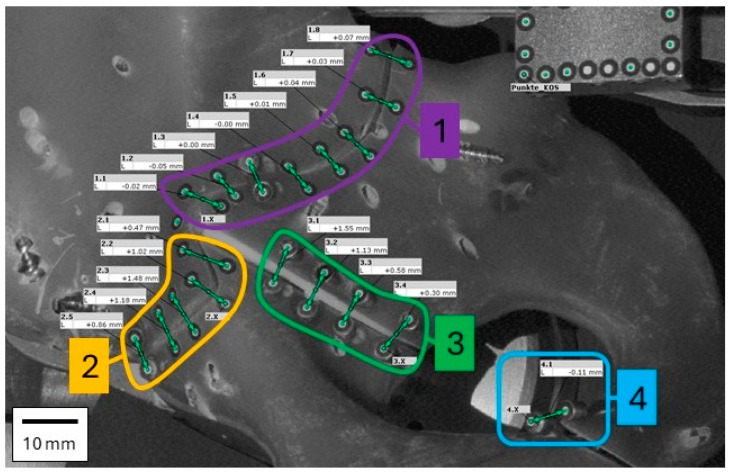
Detailed view of the software evaluation of the relative point distances (corresponds to prior photograph in Figure 1). These represent a 2D visualization of a 3D point cloud. The points at the top right are used to determine a constant coordinate system (not shown in other figures). The four mentioned zones are marked for clarity (compare with Figure 1 and Figure 5).

Pairs of optical reference markers (uncoded white markers, 1.5 mm diameter, Carl Zeiss GOM Metrology GmbH, Braunschweig, Germany) were positioned along the fracture lines posterior to the acetabulum in the following four zones: Zone 1 (8 pairs), Zone 2 (5 pairs), Zone 3 (4 pairs) and Zone 4 (1 pair) (Figure 4). These markers were captured with a stereo camera system in grayscale, and a 3D point triangulation was performed to calculate the 3D position of the markers in the defined coordinate system (using an ATOS Core 300 scanner, Carl Zeiss GOM Metrology GmbH). The 3D distance of the markers along the fracture line in zones 1–4 was measured simultaneously in the x-, y- and *z*-axis using the optical measurement system and Aramis software (version 2021, Carl Zeiss GOM Metrology GmbH). The resulting gap was zeroed at the beginning of the test and was then calculated using the following formula $\left|\vec{R}\right|=\;\sqrt{x^{2}+y^{2}+z^{2}}$.

**Figure 5:**
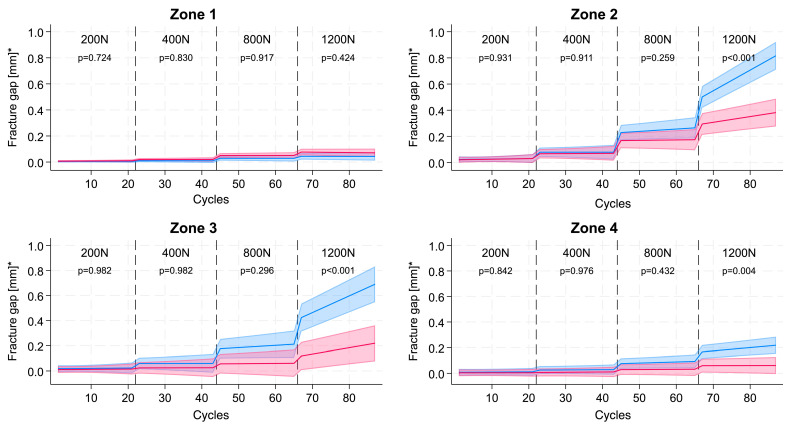
The modelled course of the mean fracture gap under load over the course of cyclic, increasing load in GRP (blue) vs. BSR (red) fracture fixation in the four different fracture zones. The *p*-values show the comparison between the slopes in each load category (* predictive marginal mean with 95% CI of the fracture gap between groups). Modelled slope and intercept comparisons confirm a significantly steeper increase in fracture gap with GRP in zones 2–4 at higher loads (≥800 N, *p* < 0.01) while remaining under 1 mm (1000 µm).

Using an electrodynamic testing machine (LTM 10, ZwickRoell GmbH & Co. KG, 89079 Ulm, Germany), loads were applied in the direction of the greatest load occurring during normal gait as determined by Bergmann [9,10,11]. According to Bergmann, the maximum load during walking is 238% of the normal body weight [10]. We assumed a body weight of 80 kg, which translates to a load of approx. 1.8 kN. Cyclical load levels of 200 N, 400 N, 800 N, and 1200 N representing partial weight bearing were applied, as this is a frequent restriction in the reported patient collective. At each load level 10 × 100 cycles were applied (with a testing frequency of 1 Hz) in the direction of maximal load, as done in prior studies [17], and after the first and last cycle of each 100-cycle set, images were taken during maximal (F_max_) and minimal (F_min_) loads. Communication between the electrodynamic testing machine and the optical measurement system was implemented using a C 2014 controller (Carl Zeiss GOM Metrology GmbH) to use electrical analog signals from the testing machine as a signal for image recording. According to the manufacturer’s specifications, the optical measurement system provides sub-50 µm accuracy (during tests according to VDI/VDE 2634, Part 3, the system showed a maximum length measurement deviation of 12 µm). The measurements made are true 3D Euclidean distances.

### 2.2. Statistical Analysis

The statistical analysis was conducted using Stata/MP 18.0 for Mac (Apple Silicon), StataCorp, College Station, TX, USA. All analyses were stratified by the four zones of the T-type acetabular fracture, i.e., Zone 1, the posterior column (transverse fracture gap), Zone 2, the anterior column (transverse fracture gap), Zone 3, the anterior/posterior column (vertical fracture gap), and Zone 4, the ischiopubic ramus (vertical fracture gap). For descriptive analysis, the course of the mean displacement (fracture gap) of the difference in all pairs within a single loading zone was shown for each zone in a scatter plot.

The course of the mean fracture gap of the pairs (aggregated before modelling) under cyclic, increasing load was then compared between the two types of fracture fixation using mixed effects linear regression with a random intercept for acetabulum number to take into account the repeated measurement nature of the data and a random slope over time. Within one load category (200 N, 400 N, 800 N, and 1200 N) a linear course of the fracture gap under non-extreme, increasing, cycling load over time was considered. As a measure of effect, the difference at the beginning of each load category as well as the difference in the slopes of the two fracture fixation techniques was presented. *p*-values for the comparison between the fracture fixation techniques were obtained for each load category using Stata’s—contrast—postestimation command. For the sensitivity analyses, the median instead of the mean displacement of the pairs was used.

Multiple measurements were obtained from each hemipelvis over repeated loading cycles, and all analyses were performed using linear mixed-effects models. Specimen identity was treated as the independent experimental unit and included as a random intercept, with loading cycle modelled as a repeated measure (random slope) to account for within-specimen correlation. Consequently, individual loading cycles were not treated as independent observations, and no pseudo-replication was performed. Statistical inference was therefore based on specimen-level variance rather than cycle count.

These analyses were repeated for the difference in the fracture gap between load and relaxation. A *p*-value of ≤0.05 was considered significant.

## 3. Results

Under increasing loads there is only very minimal displacement in all four zones up to a load of 400 N in either model. At 800 N the fracture gap in zones 2 and 3 increases slightly to 0.200 mm (200 µm) in the GRP-construct specimen; however, the difference in gap size to the BSR-construct specimen is not statistically different. At 1200 N the difference between the two fixation techniques becomes significant, the gaps of the GRP-construct specimen are statistically wider in zones 2, 3 and 4 and are especially in zones 2 and 3 when compared to the BSR-construct specimen. Post-test inspection revealed no macroscopic failure or visible deformation of any construct.

Figure 5 illustrates the modelled course of the fracture gap for GRP and BSR fixation methods under the cyclic, increasing load categories (200 N, 400 N, 800 N, and 1200 N). At low loading conditions (200 N and 400 N), the fracture gap remains small with no statistically significant differences between the slopes observed (*p* = 0.931 and *p* = 0.911, respectively). As the load increases to 800 N, a slight divergence in the fracture gap emerges, with the GRP fixation method showing a marginally larger gap compared to BSR, without a significant difference in the slopes (*p* = 0.259). At the highest load of 1200 N, a pronounced divergence is evident, with the GRP fixation exhibiting a significantly larger fracture gap than BSR in zones 2 and 3 (*p* < 0.001).

## 4. Discussion

PD is a complete separation of the hemipelvis through the acetabulum, with the ilium superiorly and the ischium and pubis inferiorly. It is considered to consist of two separate entities—acute and chronic PD, although there is considerable overlap between the two. Acute and chronic PD differ in etiology, biology and biomechanical stability and have differing treatment regimens accordingly [19]. Acute PD results from traumatic fracture and assumes no or minimal bone loss. In contrast, in chronic PD the acetabular separation is due to extensive bone loss and secondary fibrosis from osteolysis and component loosening.

The incidence of PD, both acute and chronic, has been variously reported as 1–5% after PTHA [20,21,22]. Berry reported an incidence of 0.9% of all RTHA [19,23]. Acute PD has a bimodal occurrence with a peak in the 20–30 year-old age group and a larger peak in older adults. These latter patients constitute the largest and fastest growing group of patients, most of whom sustain acute PD from low-energy trauma such as a fall from a chair or from walking height [3,24]. Older females are particularly vulnerable [25]. Other patient-related risk factors are rheumatoid arthritis, a history of pelvic radiation, Paget’s disease, obesity, and steroid therapy [26].

Iatrogenic acetabular fractures may contribute to the incidence of acute PD and have been reported with an incidence of 0.09–0.4% [27] and are associated with acetabular under or over-reaming during placement of a porous acetabular cup [21]. Patient-related risk factors for intraoperative acetabular fracture are the same as those for traumatic fracture [28]. Intraoperative acetabular fractures have not been associated with cemented acetabular cups [29]. Postoperative periprosthetic acetabular fracture identified by computerized tomography several weeks after surgery has been reported with an incidence of 6.9% [30] and 8.4% [28] and thought to represent occult fractures that occurred operatively during primary THA. Male patients account for a higher proportion of perioperative fractures, which is thought to result from their hard, sclerotic, and possibly brittle acetabular rims [28,30]. Most perioperative fractures are nondisplaced, most heal without surgical intervention [30] and most do not result in acute PD [27]. However, since most are occult, there is little data on long-term outcomes particularly for high-risk patients with poor bone quality.

Acute PD fractures from trauma, particularly distinct trauma, are most frequently transverse and T-shaped [19,31] and associated with instability of the posterior column. Factors required for a successful operative outcome with reduced risk of post-traumatic arthritis are: optimal reduction in the fracture (in particular the posterior column), restoration of pelvic bone stability and stable fixation of the acetabular component. Residual displacement of >3 mm is considered a poor radiographic outcome [32]. Integrity of the posterior column is crucial, as this is a major load-bearing region of the pelvis [24]. In older patients with multiple comorbidities and varying degrees of osteoporosis who sustain acetabular fractures, the optimum treatment regime is unclear, and each case is unique [24]. Treatment options include ORIF, ilioischial cages, and cup-cage constructs.

A joint preservation strategy using ORIF alone utilizes axial compression of the fracture site to diminish interfragmentary movement and promote mineralized rather than fibrous callus formation and denser lamellation of the bony bridge [33]. However, it is associated with a lengthy period of restricted weight bearing that can predispose to increased risk of deep venous thrombosis, pneumonia, post-traumatic arthritis and permanent loss of mobility, particularly in older adults [24]. The development of posttraumatic arthritis occurs in 12–57% of patients [34,35,36,37] requiring conversion to THA in up to 31% [38,39,40,41,42,43]. A second surgery for older adults places them at additional risk; therefore, a one-stage treatment regime that provides the necessary fracture reduction and pelvic primary stability is desirable (as was done in this fix-and-replace regime).

Ilioischial cages that bridge across gaps and defects can eventually develop metal fatigue and fail in the absence of coincident biological fixation. Studies on revision rings have shown that these perform better in older adults, which may be a result of the lower physical activity levels [44]. Long-term survivorship is noted to be better with the cup/cage construct, which is a modern, yet costly, implant utilizing a porous surface to permit osseointegration [27,45,46]. In osteoporotic bone, fracture healing and desired implant osseointegration may be delayed or impaired [4,47]. Hence, we tested a scenario where revision rings may provide a valid treatment option, as the success of these implants may be less dependent on such factors.

Our biomechanical model most closely replicates the clinical scenario found in patients with acute PD, with a potential minimal interfragmentary gap, minimal or no dislocation or bone loss. This setup differs from most prior studies by focusing on pelvic bone stability by assessing the primary stability at several points along the fracture line while under increasing load, rather than the primary stability of the bone porous acetabular cup interface, as prior studies have done [17,48,49]. A prerequisite for successful operative outcome and ultimate osseointegration is pelvic bone stability and optimal reduction in pelvic bone fragments with minimal gap size and micromotion. Fracture gaps should remain below 2 mm, since larger fracture gaps (>2 mm) can impair angiogenesis and thus hinder bone regeneration [33].

In our study design, we defined failure as a fracture dislocation >1 mm as a conservative estimate for failure, since the literature has shown poorer outcomes in acetabular fractures with fracture gaps ≥ 2 mm [50]. Our results show minimal displacement at the fracture gap at all tested loads, which should permit potential bone ingrowth and fracture healing under a partial weight-bearing scenario. Higher loads (e.g., 1600–1800 N) may reveal additional construct differences, but we deliberately limited testing to partial weight-bearing loads, to reflect realistic early postoperative conditions in this patient population. Observed differences at higher loads should be interpreted as construct-specific biomechanical behaviour, not construct failure. Although our setup did not measure the relative motion between bone and implant, the minimal displacement at the fracture site implies favourable conditions for osseointegration between the two.

## 5. Limitations

We acknowledge some limitations in our study. Our tests were performed on a Sawbone model. While the use of Sawbones models does not fully replicate the biological properties of human bone, it also represents a methodological strength by providing standardization and enabling differences to be attributed solely to the fixation technique. The model used focuses on construct-related and geometric stability in acute PD with preserved bone stock, rather than biological fracture healing or osteoporotic bone failure. Although the use of highly standardized 4th generation Sawbone models, which do not completely replicate the trabecular bone structure of (osteoporotic) bone, and repeated measurements analyzed with mixed-effects regression increases precision, the experiment was performed on a limited number of specimens and should be interpreted as an exploratory biomechanical comparison with possible conservative results rather than a definitive equivalence trial. As such, these results may not be extrapolated to severe osteoporotic bone with screw failure. To our knowledge, there is no evidence indicating the critical fracture gap size at which aseptic loosening may occur. The movement of the cup relative to the acetabulum was not measured. It remains unclear how much fracture gap motion is required for healing and how much would be excessive, potentially leading to implant failure. It is also uncertain how many loading steps or cycles must be tolerated before healing occurs. Furthermore, only a standardized T-type acetabular fracture pattern with acute PD was investigated. As a result, the results may not be extrapolated to other fracture configurations or to cases of chronic PD, which may present with different biomechanical characteristics.

## 6. Conclusions

Both fixation methods demonstrated sufficient construct stability without catastrophic failure, with minimal displacement (<1 mm) and with no significant difference in stability at the posterior column under partial weight-bearing conditions. Further clinical evaluation is required as well as comparison with other implant designs to determine the best treatment for patients presenting with acute traumatic PD and qualifying for a fix-and-replace strategy.

## Data Availability

Dataset available on request from the authors.

## Abbreviations

The following abbreviations are used in this manuscript:

PD	Pelvic Discontinuity
APD	Acute Pelvic Discontinuity
THA	Total Hip Arthroplasty
PTHA	Primary Total Hip Arthroplasty
RTHA	Revision Total Hip Arthroplasty
GRP	Ganz Reinforcement Ring with Posterior-column Plate and Anterior-column Screw
BSR	Burch-Schneider Reconstruction
ORIF	Open Reduction and Internal Fixation
